# Emerging roles of the SUMO pathway in mitosis

**DOI:** 10.1186/1747-1028-3-5

**Published:** 2008-01-24

**Authors:** Mary Dasso

**Affiliations:** 1 Laboratory of Gene Regulation and Development, NICHD/NIH, Building 18, Room 106, MSC-5431, Bethesda, MD 20892, USA

## Abstract

SUMO proteins are small ubiquitin-like modifiers found in all eukaryotes that become covalently conjugated to other cellular proteins. The SUMO conjugation pathway is biochemically similar to ubiquitin conjugation, although the enzymes within the pathway act exclusively on SUMO proteins. This post-translational modification controls many processes. Here, I will focus on evidence that SUMOylation plays a critical role(s) in mitosis: Early studies showed a genetic requirement for SUMO pathway components in the process of cell division, while later findings implicated SUMOylation in the control of mitotic chromosome structure, cell cycle progression, kinetochore function and cytokinesis. Recent insights into the targets of SUMOylation are likely to be extremely helpful in understanding each of these aspects. Finally, growing evidence suggests that SUMOylation is a downstream target of regulation through Ran, a small GTPase with important functions in both interphase nuclear trafficking and mitotic spindle assembly.

## Introduction

SUMO proteins are small ubiquitin-like modifiers that become covalently conjugated to cellular proteins. In budding yeast, proteomic experiments indicate that 300 or more proteins may be SUMOylation targets [1-4]. This post-translational modification controls multiple events, including transcription, DNA repair, DNA recombination and mitotic chromosome segregation. The three former processes were covered within recent reviews [5-11], and will not be discussed here. Rather, I will focus on evidence that SUMOylation plays a critical role in mitotic chromosome structure and segregation, and on how this pathway may be regulated during mitosis.

### SUMO proteins and their conjugation pathway

**Figure 1:**
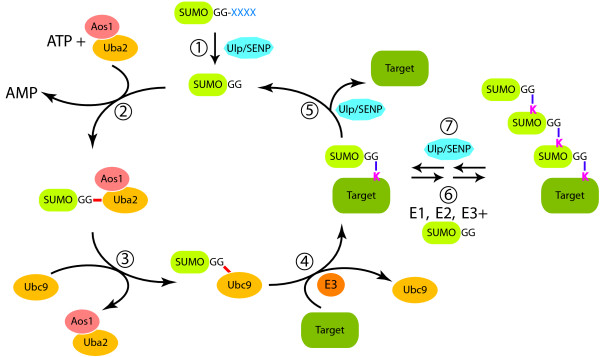
**SUMO pathway**. SUMO proteins undergo post-translational maturation, catalyzed by Ulp/SENPs, to reveal a C-terminal di-glycine motif (Step 1). Mature SUMOs undergo ATP-dependent activation, resulting in a thiolester linkage between the C-terminal di-glycine and their activating enzyme, Uba2/Aos1 (Step 2). The thiolester is transferred to their conjugating enzyme, Ubc9 (Step 3). Ubc9 acts in concert with SUMO ligases/E3 enzymes to form an isopeptide linkage between the SUMO C-terminus and an ε-amino group of a lysine within the target protein (Step 4). SUMOs can be removed from conjugated species by the action of Ulp/SENPs (Step 5). In some cases, SUMO chains can be formed through linkage of additional SUMO moieties to previously conjugated SUMOs (Step 6). While it is possible that multiple Ulp/SENPs may disassemble SUMO chains (Step 7), members of the Ulp2 family appear to be specialized for this reaction.

There is one SUMO protein in *S. cerevisiae *(Smt3p) and *S. pombe *(Pmt3), but mammalian cells typically express three SUMO paralogues (SUMO1-3) [12]. Like ubiquitin, newly translated SUMOs require cleavage to reveal C-terminal diglycine motifs (Figure 1, Step 1). After maturation, SUMO1 is ~45% identical to SUMO2 or 3, while SUMO2 and 3 are ~95% identical to each other. Where they cannot be distinguished, I will refer to SUMO2 and 3 collectively as SUMO2/3. Proteases of the Ubiquitin like protein protease/Sentrin specific proteases (Ulp/SENPs) family catalyze SUMO processing [13]. *S. cerevisiae *has two Ulp/SENPs (Ulp1p and Ulp2p/Smt4p). Ulp1p associates with the nuclear envelope [14], and is important for Smt3p maturation [15]. *S. pombe *likewise has two Ulp/SENPs (also called Ulp1 and Ulp2), while mammals have six (SENP1, 2, 3, 5, 6 and 7) [13].

Conjugation of mature SUMOs occurs through a cascade (Figure 1, Steps 2–4) containing a heterodimeric activating enzyme (E1 enzyme. Uba2/Aos1), a conjugating enzyme (E2 enzyme. Ubc9) and usually a SUMO ligase (E3 enzyme) [12]. Nomenclature of SUMO pathway enzymes in yeast and vertebrates are given in Table 1. The result of these reactions is an isopeptide linkage between the SUMO C-terminal glycine and an ε-amino group of a lysine within the target protein. The biochemistry of SUMO and ubiquitin conjugation are similar, but no enzymes act on both SUMOs and ubiquitin. On the other hand, the same E1 and E2 enzymes act for the conjugation of all mammalian SUMO paralogues. SUMO-conjugated species are deconjugated by Ulp/SENPs (Figure 1, Step 5), rendering this modification highly dynamic. Smt3p, SUMO2 and SUMO3 can form chains, primarily through a conserved acceptor lysine [16-18] (Figure 1, Step 6). SUMO1 can form chains *in vitro *through other lysine residues [19], although SUMO1 chains have not been demonstrated *in vivo*. A subset of Ulp/SENPs is specialized for dismantling of SUMO chains (Figure 1, Step 7): In budding yeast, Ulp2p is predominantly nuclear [20]. Many phenotypes of *ulp2 *mutants arise from their inability to disassemble Smt3p chains, suggesting that it is critical for this reaction [17]. A related protein, SENP6, has been implicated in chain editing in mammalian cells [21].

**Table 1:** SUMO Pathway Enzymes

**Enzymatic activity**	**Vertebrate**	***S. cerevisiae***	***S. pombe***
SUMO paralogues	SUMO1	Smt3p	Pmt3p
	SUMO2		
	SUMO3		
SUMO protease	SENP1-3, SENP5-7	Ulp1p	Ulp1
		Ulp2p/Smt4p	Ulp2
Activating Enzyme (E1)	Uba2/SAE2 +Aos1/SAE1	Uba2p+Aos1p	Uba2/Fub2 + Rad31
Conjugating Enzyme (E2)	Ubc9	Ubc9p	Hus5p
SP-RING SUMO ligases (E3)	PIAS1	Siz1p	Pli1
	PIAS3	Siz2p/Nfi1p,	
	PIASxα		
	PIASxβ		
	PIASy		
	Mms21	Mms21p	Nse2p
		Zip3p	
	Zimp7*		
	Zimp10*		
Other SUMO ligases (E3)	RanBP2	(None known)	(None known)
	Pc2		

*Related to PIAS/Siz family, but SUMO ligase activity not been demonstrated *in vitro*.

There are multiple SUMO E3 enzymes, and it is likely that their specificity plays a significant role in determining the spectrum of SUMOylated species. A conserved group of SUMO E3 enzymes found in all eukaryotes possess variant RING-finger like domains (SP-RINGs). This class of E3 enzymes are called Siz (SAP and miz-finger domain) proteins in yeast and PIAS (protein inhibitor of activated STAT) proteins in vertebrates (reviewed in [22]). Budding yeast Siz1p and Siz2p proteins are required for the bulk of Smt3p conjugation [23,24]. The SUMO ligase activity of other SP-RING proteins, Mms21p and Zip3p, are required for DNA repair [25-27] and meiotic synaptonemal complex assembly [28], respectively. Vertebrates express five PIAS proteins (PIAS1, PIAS3, PIASxα, PIASxβ and PIASy), which have been implicated in a broad variety processes, including signal transduction, gene expression and genome maintenance. [29]. Humans also express a homologue of Mms21p [30] and two additional SP-RING proteins, hZIMP7 and hZIMP10 [29].

There are other E3 enzymes in vertebrates that have no obvious homologues in yeast. Vertebrate-specific E3s include Pc2, a polycomb group protein [31], and RanBP2, a large nuclear pore protein that localizes to the cytoplasmic face of the nuclear pore complex (NPC) [32]. No mitotic role of Pc2 has been established. However, the interactions and mitotic behavior of RanBP2 has been intensively studied. RanBP2 possesses a domain called the IR domain, which is comprised of two short, tandemly repeated sequences (around 50 residues), separated by a 24-residue spacer. IR-containing fragments of RanBP2 have SUMO ligase activity *in vitro *[32]. This domain is also the site of assembly for a complex that contains Ubc9 and the SUMO1-modified form of RanGAP1 (RanGAP1•SUMO1) [33], which will be called the RRSU complex. RanGAP1 is the activating protein for the abundant small GTPase Ran [34]. Ran is required for many cellular functions, including nucleocytoplasmic trafficking, spindle assembly and cell cycle control. RanBP2 possesses four Ran-binding domains that enhance RanGAP1-mediated GTP hydrolysis by Ran.

### Genetic links of SUMOylation and mitosis

The link between SUMOylation and mitosis was established very early in the history of this field. Even before the discovery of SUMO proteins themselves, it was known that budding yeast Ubc9p is essential for degradation of B-type Cyclins [35], key mitotic regulators that are destroyed through ubiquitination at anaphase onset. Moreover, SMT3 was isolated in yeast screens for temperature-sensitive mutants defective in chromosome segregation [36], and for suppressors of mutations of *mif2*, a homologue of the vertebrate centromeric CENP-C protein [37]. Ongoing characterization of mutants lacking SUMO pathway components has confirmed that many of them have important roles in mitosis.

Smt3p, Uba2p, Aos1p and Ubc9p are encoded by essential genes in budding yeast, consistent with the central role of SUMO pathway in many aspects of cell physiology [38-40]. Δsmt3 strains of *S. cerevisiae *arrest in early mitosis as large budded cells with short spindles [38]. Progression through mitosis requires the anaphase promoting complex/cyclosome (APC/C), a ubiquitin ligase that is responsible for controlled degradation of B-type Cyclins and other substrates at anaphase onset. The APC/C is controlled through the spindle assembly checkpoint (SAC), a regulatory pathway that monitors spindle formation and inhibits APC/C until all chromosomes are correctly attached and aligned on the metaphase plate. Mitotic arrest of Δsmt3 cells does not reflect anaphase inhibition through the SAC [38]. Rather, it results from their inability to appropriately activate the APC/C after the SAC is turned off. A similar defect in APC/C activation is observed in Δubc9 cells [38]. Finally, temperature-sensitive uba2 mutants (uba2-*ts*) show pronounced hypersensitivity to microtubule destabilizing drugs and early mitotic arrest with short, frequently misaligned spindles at the restrictive temperature [40].

In fission yeast, the gene encoding Pmt3p is not essential, nor are the genes encoding the homologues of Uba2p or Ubc9p, although mutants lacking any of these proteins show very slow growth. pmt3Δ cells show a spectrum of phenotypes, which are indicative of problems in mitotic chromosome structure or segregation errors [41]. These defects include high frequency loss of mini-chromosomes and a cut (cell untimely torn) phenotype. Similar chromosome segregation phenotypes are found in strains with mutations in *S. pombe *Aos1 [42] and Ubc9 [43] homologues. In a manner that is perhaps related, transgenic mice lacking Ubc9 show aberrant chromosome structures in mitosis, including hypercondensation and breakage, as well as a high rates of missegregation and polyploidy [44]. These defects result in early embryonic lethality in Ubc9-deficient mouse embryos (<E7.5).

Mutations in yeast genes encoding SUMO ligases do not cause strong mitotic phenotypes. This observation might either suggest that E3 enzymes function redundantly for key substrates, or that the low level of conjugation obtained by Ubc9 alone is sufficient for mitosis. While most Smt3p conjugation is lost in mutants lacking Siz1p and Siz2p, such double mutants remain viable and grow without pronounced defects in the absence of 2 μm circles [45]. 2 μm circles are stable extrachromosomal elements that are carried in multiple copies by most budding yeast strains, although they are not necessary for viability.

There is more direct evidence of mitotic requirements for individual E3 enzymes in vertebrates: PIASy is required for chromosome segregation in Xenopus egg extracts [46] and mammalian tissue culture cells [47]. Surprisingly, transgenic mice lacking PIASy are viable, morphologically normal and fertile [48]; this finding suggests that other PIAS proteins may complement SUMOylation deficiencies in PIASy^-/- ^mice, or that cells are able to bypass this requirement in vivo through other mechanisms. Separately, numerous observations indicate that the vertebrate RanBP2 protein has an important mitotic role, particularly in promoting the correct attachments of kinetochore microtubules (kMTs) to kinetochores [49-52]. However, it has not been demonstrated that the capacity of RanBP2 to promote such attachments requires its activity as a SUMO ligase.

Finally, SUMO proteases are important within mitosis. Budding yeast *ulp1 *mutants show cell cycle delays at the G_2_/M boundary and elevated chromosome mis-segregation [15], and these defects are exacerbated by the presence of 2 μm circles [53]. Budding yeast Ulp2p is not required for vegetative growth, although it is important for meiosis, chromosome segregation and recovery from DNA replication and spindle assembly checkpoint arrests [20,54]. During mitosis, *ulp*2 mutants show precocious loss of centromeric cohesion [55] and defects in rDNA condensation [20]. Like its budding yeast counterpart, *S. pombe *Ulp1p localizes to the nuclear envelope [56], and *ulp1*Δ strains show pronounced growth defects, resembling mutants deficient in the fission yeast Aos1p or Ubc9p homologues. Moreover, fission yeast lacking Ulp1p develop elongated and irregular morphologies in comparison to wild-type cells, with frequent mis-localization of the nucleus within the cell and increased incidence of multiple septations [56].

### Mitotic targets of SUMOylation

**Figure 2:**
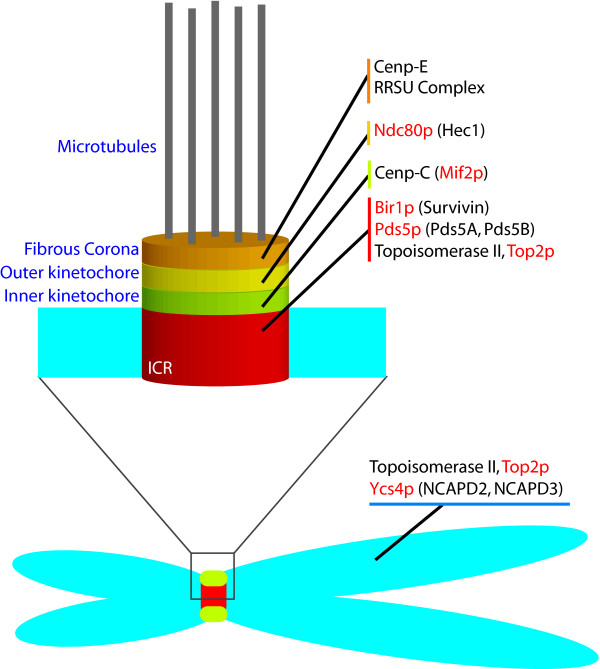
**Mitotic chromosomal SUMOylation substrates**. The distribution of SUMOylation substrates is schematically represented, based on the localization of the vertebrate homologues. (The localization reflects the bulk of each protein on mitotic vertebrate chromosomes, not specifically the SUMOylated forms.) The names of vertebrate proteins are indicated in black, while corresponding budding yeast proteins are given in red. In cases where SUMOylation has not been confirmed in both vertebrates and yeast, parentheses indicate the homologue for which demonstration is lacking. This representation does not include many proteins identified in proteomic screens [1-4] whose conjugation has not been independently verified, nor yeast proteins without obvious vertebrate homologues (e.g., Ndc10p, Cep3p [63]). Confirmed SUMOylation substrates associated to mitotic chromosomes (e.g., Histones [95]) are similarly not represented if the timing of their modification has not been demonstrated within the cell cycle.

Mitotic targets for SUMOylation have been documented from yeast to vertebrates. I will use budding yeast nomenclature for particular targets, unless explicitly stated otherwise. Both vertebrate and budding yeast names are provided in Figure 2 for substrates whose mitotic conjugation has been confirmed. In many cases, the role of SUMOylation in regulating individual targets is poorly understood, frequently not extending beyond their initial identification in broad proteomic screens [1-4]. Despite these problems, it is worth discussing target proteins because their variety suggests that SUMOylation is required for numerous aspects of mitosis. This review will consider three classes mitotic targets: namely, proteins with a general role in mitotic chromosome structure, proteins with specific roles at the kinetochore and cytosolic SUMOylation targets.

#### Mitotic Chromosome Structure: Condensin, Cohesin and Topoisomerase II

The Condensin and Cohesin complexes are among the most intriguing mitotic SUMOylation targets. Both complexes contain Structural Maintenance of Chromosomes (SMC) proteins. SMCs are large proteins that form rod-shaped structures through antiparallel coiled-coil interactions, with an ATP-binding globular domain at one end and a hinge domain at the other [57]. Eukaryotes have six SMC family members, which form heterodimers in specific combinations: Smc1p and Smc3p are constituents of Cohesin complexes, which maintains sister chromatid cohesion until the onset of anaphase. Smc2p and Smc4p constitute the core of Condensin complexes, which maintain the condensed structure of mitotic chromosomes. SMC proteins within both of these complexes (Smc1p, Smc3p, Smc4p) have been identified as SUMOylation substrates in budding yeast through proteomic screens [1,4].

Smc1p and Smc3p associate with non-SMC subunits Scc1p and Scc3p to form Cohesin [57]. Like Smc1p and Smc3p, Scc1p was identified in proteomic searches for yeast SUMOylation targets [1,4]. In vertebrates, much of the Cohesin is released from chromosome arms during prophase through the action of the Plk1 and Aurora B kinases. The remaining vertebrate Cohesion is concentrated in the pericentromeric region. In both vertebrates and yeast, all residual Cohesin is released at anaphase through cleavage of the Scc1p by a specific protease, called Separase [57]. Before anaphase, Separase is inhibited through interaction with Securin, a key APC/C target, whose degradation allows Separase activity. The Pds5p protein associates with Cohesin and modulates its binding to chromosomes. Yeast with temperature sensitive mutations in PDS5 (*pds5-1*) can establish but cannot maintain cohesion, and undergo precocious sister chromatid separation at the restrictive temperature [58]. Pds5p is SUMOylated in a cell cycle-dependent manner, beginning in S-phase and peaking immediately prior to anaphase onset [58]. ULP2 acts as a high-copy suppressor of the cohesion defect in *pds5-1 *and other strains with *ts *alleles of PDS5, but not *pds5 *null alleles. Conversely, overexpression of Siz1p significantly enhances the sensitivity of *ts pds5 *strains. Consistent with these results, increased Ulp2p levels decrease Pds5p SUMOylation, while elevated Siz1p enhances Pds5p modification. These findings suggest that Pds5p interacts with the Cohesin complex to increase the strength of sister chromatid cohesion, and that SUMOylation disrupts this interaction to facilitate cohesion release. It is not yet clear how Pds5p SUMOylation works in parallel with Scc1p proteolysis by Separase, nor how SUMOylation of other Cohesin subunits controls Cohesin function.

Within the Condensin complex, Smc2p and Smc4p associate with non-SMC subunits Ycs4p, Ycs5p and Brn1p. A genetic relationship between Smt3p and Condensin was originally suggested by the finding that Ulp2p overexpression suppresses the *smc2-6 *allele, and from the observation that *Δulp2 *yeast are deficient in targeting of the Condensin complex to mitotic chromatin, particularly to rDNA [20]. In addition to Smc4p, Ycs4p and Brn1p were identified as potential SUMOylation targets in yeast [1,4]. Functional ramifications of SUMOylation have not been demonstrated for individual Condensin subunits, with the exception of Ycs4p. A fraction of Ycs4p becomes SUMOylated immediately prior to mitosis, with more highly SUMOylated forms peaking during anaphase [59]. The SUMOylation of Ycs4p during anaphase depends upon Cdc14p, a phosphatase that promotes Condensin association with rDNA. These findings might suggest that Cdc14p promotes SUMOylation of Ycs4p, which in turn facilitates Condensin localization to the rDNA during anaphase. On the other hand, inactivation of Ulp2p results in reduced of Ycs4p and other Condensin subunits with the nucleolus and inefficient rDNA segregation [20,59], arguing against a simple antagonistic relationship between these two enzymes in Ycs4p targeting, and suggesting that this pathway has considerable complexity.

Finally, Topoisomerase II is a major target of SUMOylation in both budding yeast and vertebrates. In yeast, ulp2Δ strains show precocious loss of centromeric cohesion in a manner that is not dependent upon Scc1p cleavage or re-distribution of the Cohesin complex [55]. This loss is suppressed by overexpression of Top2p, the yeast Topoisomerase II homologue. Top2p is itself SUMOylated, and *top2 *mutants that lack SUMO conjugation sites are efficient suppressors of the cohesion defect in ulp2Δ strains. These findings collectively suggest that Top2p plays an important role in centromeric cohesion, which is down-regulated through its SUMOylation [55]. In mitotic Xenopus egg extracts, Topoisomerase II is a major substrate for PIASy- and chromatin-dependent conjugation with SUMO2/3 [46,60]. A small fraction of the Topoisomerase II assembled onto mitotic chromosomes in Xenopus egg extract is resistant to high salt extraction. Inhibition of SUMO modification by a dominant-negative Ubc9 mutant (dnUbc9) dramatically increases the amount of unmodified Topoisomerase II retained within this population. At the same time, egg extracts treated with dnUbc9 fail to segregate their chromosomes in anaphase, consistent with the possibility that SUMO-dependent re-modeling of Topoisomerase II promotes release of the tightly bound population and of cohesion.

#### Centromeres and kinetochores

Centromeres are epigenetically-specified chromatin domains on each chromosome that facilitate the accurate segregation sister chromatids during mitosis [61]. Kinetochores are proteinaceous structures that assemble on the centromere of each mitotic sister chromatid. They serve as sites of spindle microtubule (MT) attachment. The kinetochore fibers (k-fibers) that link mammalian kinetochores to spindle poles contain both MTs that are directly attached to the kinetochores at their plus ends (kMTs) and MTs that are not [62]. Kinetochore-kMT attachment is monitored through the SAC [61]. The inner centromeric region (ICR) lies between sister centromeres, and contains proteins required for the regulation of sister chromatid cohesion and kMT attachment. Multiple functions of SUMOylation have been proposed within mitotic centromeres and kinetochores, and recent reports have suggested that many proteins within these domains are SUMOylation targets.

In mitotic Xenopus egg extracts, the ICR contains an abundance of SUMO2/3-conjugated species that are modified in a PIASy-dependent manner [46]. Proteomic analysis in budding yeast has identified numerous kinetochore- or centromere-associated SUMOylation targets by mass spectrometry, including: Bir1p [4], Cbf1p [1,4], Cbf5p [4], Mcm21p [4], Ndc10p [4], Ndc80p [1], Sli15p [4] and Slk19p [1]. Montpetit et al. subsequently confirmed and analyzed the SUMOylation of Ndc10p, Bir1p and Ndc80p, as well as SUMOylation of Cep3p [63]. Ndc10p and Cep3p are subunits of centromeric DNA binding factor 3 (CBF3), a four-protein complex that binds to an essential element within centromeric DNA [64]. Bir1p and Sli15p link CBF3 to MTs *in vitro*, and may sense tension to activate the Ipl1p kinase in the vicinity of syntelic attachments [65]. The vertebrate homologues of Bir1p, Sli15p and Ipl1p are Survivin, INCENP and the Aurora B kinase, respectively [66]. Survivin, INCENP and Aurora B constitute the chromosomal passenger complex (CPC) in combination with Dasra-B [66]. The CPC is a key player in the spatial and temporal ordering of mitosis, which controls kinetochore-MT interactions, sister chromatid cohesion and cytokinesis. Ndc10p, Cep3p and Bir1p are deSUMOylated by Ulp2p in response to nocodazole, suggesting that their modification is regulated through SAC activation [63]. Mutants that eliminate Ndc10p modification cause chromosome instability, mislocalization of Ndc10p from the mitotic spindle, abnormal anaphase spindles, and a loss of Bir1p SUMOylation, suggesting an important role for SUMO conjugation in yeast kinetochore function [63].

Interestingly, the fission yeast of Aurora kinase Ark1p interacts with two RING-finger proteins that possess N-terminal SUMO interaction motifs (SIMs), called Rfp1p and Rfp2p [67]. These proteins and their budding yeast homologues recognize SUMOylated proteins, and heterodimerize with Slx8p, another RING-finger protein, to form a functional ubiquitin ligase [67-69]. The demonstration of this ubiquitin ligase activity has suggested a novel paradigm for SUMO-directed destruction of proteins through the ubiquitin-proteasome system. Given the physical association of Ark1p with Rfp1p and Rfp2p, it is attractive to speculate that CPC SUMOylation may direct the ubiquitination of ICR proteins, possibly including CPC members themselves.

Centromere associated protein C (CENP-C) is a vertebrate homologue of the budding yeast Mif2p protein [37]. CENP-C binds to alpha satellite DNA within centromeres [70]; it is required for the correct assembly of inner kinetochores, as well as checkpoint signaling and accurate chromosome segregation [71]. A temperature-sensitive CENP-C mutant cell line (*ts4-11*) derived from chicken DT40 cells, shows metaphase delay, but eventually proceeds through mitosis with chromosome missegregation and undergoes arrest within G_1 _phase [72]. This cell cycle arrest phenotype was utilized to screen for human cDNAs that could rescue *ts4-11 *cells, and SUMO1 was identified within this screen. This finding was interesting in light of the genetic relationship between MIF2 and SMT3 in budding yeast [37], as it suggest that SUMOylation regulates CENP-C in a manner that is conserved between vertebrates and fungi. While CENP-C can be SUMOylated under *in vitro *conditions [73], it should be noted that the capacity of SUMO1 to rescue *ts4-11 *cells may equally reflect the modification of other inner kinetochore proteins that interact with CENP-C.

In the outer kinetochore, both Ndc80p and the vertebrate centromere associated protein E (CENP-E) have been strongly implicated as SUMOylation targets. The Ndc80 complex is a conserved set of kinetochore proteins that consists of Ndc80p, Nuf2p, Spc24p, and Spc25p [74,75]. This complex is essential for metaphase chromosome alignment and anaphase chromosome segregation. Ndc80p is SUMOylated throughout the cell cycle, but this modification is not responsive to the SAC and its functional significance is not clear [63]. SUMOylation of Ndc80p's vertebrate homologue, Hec1, has not yet been reported. CENP-E is a plus end-directed microtubule motor of the kinesin superfamily that localizes to the outer plate of the kinetochore and fibrous corona [76,77]. It is important for the congression of chromosomes with single unattached kinetochores to the metaphase plate [78], for the maintenance of bipolar attachment of microtubules to kinetochores, for generation of tension across sister kinetochores and for the SAC [79,80]. Matunis and colleagues have recently shown that suppression of mitotic SUMOylation in HeLa cells by overexpression of SENP2 leads to a chromosome segregation defect through disruption of CENP-E targeting to kinetochores [81]. They further observed CENP-E itself is both a SUMO2/3 substrate and polySUMO2/3 binding protein. The latter activity was particularly critical, since mutation of a SUMO2/3 interacting motif (SIM-2/3) blocked kinetochore recruitment of CENP-E.

Finally, as discussed above, vertebrate RanGAP1•SUMO1 and Ubc9 bind RanBP2 [82,83], forming a RanBP2/RanGAP1•SUMO1/Ubc9 complex (RRSU complex). The SUMOylation of RanGAP1 is indispensable for assembly of the RRSU complex [82], which remains stable throughout the cell cycle [50]. The RRSU complex is targeted to outer kinetochores or fibrous corona in a MT-dependent fashion [50], and plays an important role in k-fiber assembly [49]. Under conditions where RRSU complex targeting is disrupted, kinetochores fail to maintain discrete end-on attachments to single k-fibers and showed a resultant elevation in chromosome mis-segregation [49]. Interestingly, there is substantial variability between vertebrate species and cell types in the amounts of RRSU recruited to kinetochores, with commensurate variability in the extent of chromosome mis-segregation caused by its displacement. [84]. The assembly of the RRSU complex appears to be is restricted to vertebrates, since the IR domain is not found in RanBP2 homologues within flies, worms or fungi [34]. In some other species, particularly plants, RanGAP1 is be targeted to spindles and kinetochores through mechanisms that are not dependent upon SUMOylation [85].

#### Non-chromosomal mitotic SUMOylation targets: Septins and hNinein

Septins are conserved GTP-binding proteins that link cellular membranes with the MT and actin cytoskeletons [86]. They polymerize to 10-nm filaments that serve as organizational scaffolds and that restrict diffusion between different membrane domains. Johnson and Blobel [87] identified Septins Cdc3p, Cdc11p and Shs1p as targets that give rise to the most abundant Smt3p-conjugated species during mitosis in budding yeast. This modification is highly controlled in both time and space: Conjugated Septins appear just before anaphase onset and disappear abruptly at cytokinesis, and only Septins on the mother cell side of the bud neck become modified. It is interesting to speculate that this asymmetry may be related to the polarized distribution of kinases and other cell cycle regulators involved in the budding yeast morphogenesis checkpoint, which triggers cell cycle arrest in response to insults affecting the actin or septin cytoskeleton [88]. As discussed below, the Septin SUMOylation pattern reflects tightly regulated re-localization of both conjugation and deconjugation enzymes [89]. While SUMOylation of metazoan Septins has not been reported, it has been demonstrated that *Drosophila *Septins can interact with components of the SUMO conjugation machinery *in vitro*, and that these components are re-localized during mitosis in a manner that would allow association *in vivo *[90].

Construction of a SUMOylation site triple mutant that eliminated conjugation of Cdc3p, Cdc11p and Shs1p abolished almost all mitotic SUMOylation at the bud neck, and drastically decreased the overall level of SUMOylation within G_2_/M phase budding yeast cells [87]. This triple mutant was unable to correctly disassemble of Septin filaments, and thus retained persistent Septin rings from previous divisions. This phenotype demonstrates that SUMO conjugation is important for Septin ring dynamics during the cell cycle. Despite these drastic changes in SUMOylation patterns, however, the triple SUMOylation mutant grew without defect, showed no sensitivity to stress conditions, and did not exacerbate the phenotype of a *uba2-ts10 *strain. The two other Septins expressed during vegetative growth, Cdc10p [3] and Cdc12p [1], have subsequently been found as SUMOylation targets within proteomic analysis. It is possible that low levels of mitotic SUMOylation of these Septins may have compensated for the absence of Cdc3p, Cdc11p and Shs1p modification within the triple mutant. This idea might be consistent with the fact that the triple mutant was synthetically lethal at 25°C with a *cdc12-1 *temperature sensitive allele.

Finally, there is some indication that SUMOylation plays a role at centrosomes. hNinein is a human centrosomal protein involved in MT nucleation and anchoring at the centrosome; it binds SUMO1, can be SUMOylated *in vitro*, and may also be conjugated *in vivo *[91]. Overexpression of SUMO1 can cause the recruitment of hNinein to foci within interphase nuclei, along with Pericentrin, a protein that anchors regulatory and structural molecules to centrosomes, and γ-tubulin, the primary mediator of centrosomal MT nucleation. Pericentrin also interacts with CHD3/ZFH [92]. CHD3/ZFH is a member of the chromodomain-SWI/SNF helicase family, and it is also a SUMO-binding protein [93].

### Regulation of SUMOylation within mitosis

Mitotic SUMOylation is likely to be highly regulated, both spatially and temporally. Intriguingly, there are several links of this regulation to the Ran GTPase pathway [34]. As mentioned above, RanGAP1 is cytoplasmic during interphase. Vertebrate RanGAP1 it is targeted to the NPC in a SUMO1-dependent fashion through binding to RanBP2 within the RRSU complex. Ran's sole nucleotide exchange factor, RCC1, binds chromatin throughout the cell cycle. The distribution of Ran's regulators leads to relatively high concentrations of Ran-GTP in interphase nuclei, and in the vicinity of mitotic chromatin. Local Ran-GTP levels provide cues required for correct spatial and temporal organization of cells. The main effectors of this pathway are a family of Ran-GTP-binding nuclear transport receptors that are collectively called karyopherins. Karyopherins can associate with interphase NPCs and freely traverse them in a Ran-independent manner. They interpret the high relative levels of Ran-GTP within nuclei to direct compartment-specific loading or unloading of cargo: Import receptors (Importins) bind to their cargo in the cytoplasm, traverse the NPC and dissociate upon Ran-GTP binding. Export receptors (Exportins) bind their cargo inside nuclei in ternary complexes that contain Ran-GTP. After passage through the NPC, export complexes dissociate upon Ran-GTP hydrolysis.

During mitosis, the Exportin Crm1 directs in RRSU localization to kinetochores [49,50]. Crm1 binds kinetochores in a manner that requires neither Ran-GTP nor MTs. However, inhibition of Crm1 ternary complex formation by leptomycin B (LMB), a highly specific chemical inhibitor, blocks kinetochore recruitment of RRSU in HeLa and U2OS cells [49]. Kinetochores of LMB-treated cells show increased tension, and frequently associate with continuous microtubule bundles that span their centromeres, indicating that their k-fibers do not maintain discrete end-on attachments to individual kinetochores. Since the RRSU complex contains both Ubc9 and RanBP2, a highly active E3 enzyme *in vitro*, it is attractive to speculate that these defects may reflect an inability of these enzymes to appropriately modify kinetochore-bound targets. On the other hand, it is unclear whether the RRSU acts as an E3 enzyme *in vivo*, since analysis using purified domains of RRSU components has suggested that incorporation of Ubc9 into the complex inhibits its E2 activity [33]. It is possible that the fragments used for *in vitro *studies may not accurately reflect the interactions of the full-length proteins. A more intriguing idea might be that this inhibition occurs *in vivo *but is released by some signal(s), thus subjecting SUMOylation by RRSU to biological control.

Karyopherins also regulate budding yeast Ulp1p association to the NPC. NPC localization of Ulp1p is not required for its enzymatic activity, but rather restricts its action on nucleoplasmic substrates and promotes efficient deconjugation of proteins at the NPC [14]. The NPC localization of Ulp1p is maintained through targeting sequences within the N-terminus of Ulp1p that interacts with two Importins, Pse1p and Importin-α/β [94]. The binding of Ulp1p to Pse1p or Importin-α/β is unusual because Ulp1_1–404 _is not released from either receptor *in vitro *by Ran-GTP [94], and it has been suggested that the refractory nature of this association may be important for the stability of NPC localization. Despite the stability of such interactions, they must be remodelled at the end of mitosis to allow Ulp1p-mediated deconjugation of cytoplasmic substrates, particularly Septins. It appears that these two karopherins have a complex and perhaps antagonistic roles in this process [89]: Ulp1p mutants that cannot bind Importin-α/β target aberrantly to the Septin ring in large budded cells, suggesting that Importin-α/β normally antagonizes recruitment of Ulp1 to the bud neck. Pse1p is required for this Ulp1p recruitment to the Septin Ring, and it appears that the alterations in the association of Pse1p to the NPC may be primarily responsible for allowing Ulp1p to act on Septins.

Finally, conjugation through PIAS family proteins is regulated in mitosis. For Siz1p in budding yeast, this is again accomplished through the activity of Karyopherins [89]. Importin-α/β imports Siz1p into the nucleus during interphase. Siz1p is phosphorylated in mitosis, and this may allow its export from the nucleus by the Exportin Msn5p, followed by recruitment to the Septin ring, where it mediates Septin SUMOylation. In vertebrate systems, nuclear compartmentalization is not likely to play the same role in controlling mitotic SUMOylation because vertebrates undergo open mitosis. In metazoans, it has been demonstrated that PIAS proteins are controlled through their differential recruitment to mitotic chromosomes [46]. Specifically, PIASy concentrates on mitotic chromosomes in *Xenopus *egg extracts; PIASxα shows a lower level of accumulation, but other PIAS proteins are not recruited to mitotic chromatin. Other PIAS proteins do not recruit Ubc9 onto the chromatin at levels comparable to PIASy, nor do they restore the capacity of PIASy-depleted extracts to modify Topoisomerase-II and other chromatin-bound targets. Mechanisms that may underlie the specificity of PIASy recruitment will obviously be important topics for further investigation.

## Summary

The SUMO pathway has been implicated in several aspects of mitosis, including chromosome structure, cell cycle progression, kinetochore function and cytokinesis. While these findings are clearly intriguing, much remains to be understood. Questions of particular interest will include:

1. A large number of potential SUMOylation targets have been identified through proteomic screens. While it is too soon to predict that all of these proteins are SUMOylated under physiological conditions, it seems likely that the majority of them will be genuine targets. It will be a major task to validate their status as targets, to determine the circumstances under which they are modified, and to find whether such modification is conserved between species.

2. Even for those substrates that have been well-documented as mitotic SUMOylation targets, we have almost no understanding of how this modification alters their function at a molecular level. It is likely that several paradigms will emerge, including the use of SUMOylation to direct target localization, to alter protein-protein interactions and to select targets for ubiquitin-mediated degradation. Discovering these mechanisms should offer fascinating examples of biological regulation.

3. Finally, it is attractive to speculate that the modification of proteins that mediate a variety of mitotic processes may be coordinated, in order to facilitate the temporal and spatial organization of these processes with respect to each other. As we understand the role of SUMOylation for individual targets, it will therefore be of great interest to examine whether and how these targets are linked to each other. Equally, it will be important to understand how this pathway fits into better defined regulatory schemes featuring mitotic kinases and controlled protein degradation.
